# Antibiotic-Free Nanoplasmids as Promising Alternatives for Conventional DNA Vectors

**DOI:** 10.3390/vaccines10101710

**Published:** 2022-10-13

**Authors:** Negar Seyed, Farnaz Zahedifard, Sima Habibzadeh, Roya Yousefi, Mahya Sadat Lajevardi, Elham Gholami, Sima Rafati

**Affiliations:** Immunotherapy and *Leishmania* Vaccine Research Department, Pasteur Institute of Iran, Tehran 1316943551, Iran

**Keywords:** DNA vaccine, antibiotic-free plasmids, nanoplasmid

## Abstract

DNA vaccines with their extraordinary properties are the best choice as vectors for subunit vaccines but are not in compliance with safety regulations, mainly because of the antibiotic resistance genes on their backbone. New generations of plasmids with minimum bacterial backbones are now developed as promising alternatives to pass the safety rules and be replaced for conventional plasmids. Here we have compared the nanoplasmid (with RNA-out selection system and professional HTLV-1 containing promoter) and the conventionally used pcDNA plasmid, as regards the transfection efficiency. The EGFP gene was cloned in both pcDNA-3.1^+^ and NTC9385R-MSC and transfected into COS-7 cells for expression evaluation by flow cytometry. Meanwhile, qPCR was used to analyze the EGFP mRNA copy numbers. It was concluded that the nanoplasmid, with its extraordinary properties, can be a tempting alternative to conventional pcDNA in equal or equimolar concentrations for vaccine design. These promising results can put DNA vaccines back into focus, especially regarding diseases controlled by robust cellular immune responses.

## 1. Background

Vaccines are undoubtedly among the most important medical interventions that have helped prevent many infectious diseases such as smallpox, measles, rubella, and polio, making a substantial contribution to the life-expectancy increase during recent centuries. Almost all licensed vaccines that are currently used in general vaccination programs induce neutralizing antibodies in humans. Therefore, most of the infectious diseases which are protected by humoral immunity are well controlled by killed or live attenuated vaccines which induce long-lasting antibodies in the host. Nevertheless, for some infectious diseases, such as AIDS, malaria, tuberculosis, leishmaniasis and many vector-borne diseases, there is still no preventive vaccine available on the market because the complexity of host-pathogen interactions is not well understood. In other words, antibody response alone is not protective in these infections and the intervention of the cellular immune system is needed to prevent the disease. Therefore, unravelling the underlying host-pathogen interaction is a prerequisite for a protective vaccine to be developed. Advanced technologies such as CRISPR/Cas9 for the production of non-revertible live attenuated vaccines by targeted deletion of virulence genes as well as DNA- and RNA-based subunit vaccines, are now the avant-garde innovative technologies for vaccine development against cellular immunity dependent infections.

DNA vaccines have extraordinary advantages: the protein is produced in its original format with the same post-transcriptional modifications inside the cells, robust cellular and humoral responses are induced, DNA is thermo-stable and cold-chain independent storage makes the vaccines suitable for tropical regions, There is no immune response against the plasmid backbone contrary to live viral or bacterial vectored vaccines and the DNA constructs are safer than the live attenuated pathogen [1,2]. There is also an intrinsic adjuvant effect of the DNA vaccine which originates from the bacterial backbone of the plasmid. In nature, bacterial plasmids carry un-methylated CpG motifs that are recognized via innate immune receptors. Downstream signaling by the receptors then results in activation and transmigration of transcription factors into the nucleus which ends in strong synapsis formation between dendritic cells and naïve T cells to polarize Th1 responses [3,4,5]. In addition, the stability of DNA plasmids grants long lasting production of the protein along with a more sustained Th1 response [6]. The latter effect is IL-12 dependent which is the direct result of CpG motif stimulation of innate immunity [7]. 

Despite remarkable advantages to protect against infectious diseases, DNA vaccines are restricted by regulatory authorities. The major safety concern is the antibiotic resistance gene used as selection marker on the plasmid backbone. This gene can be transferred to other bacteria by horizontal gene transfer which can spread the resistance particularly against clinically useful antibiotics. Furthermore, residual antibiotic in the final product during in vitro production steps, could induce allergic reactions e.g., against beta-lactam antibiotics. Plasmid DNA integration into chromosomal DNA is also a real threat which necessitates >85% pure circular DNA injection. Besides these safety concerns, some intrinsic technical problems have hindered general human use. The most important of all is the large size of the conventional plasmids which is inversely proportional to the effective transfection and further migration toward the nucleus. Second, is the reduced target gene expression efficiency in eukaryotic cells due to heterochromatin formation on the bacterial backbone or small inhibitory RNAs generated from the bacterial backbone in eukaryotes [8,9,10].

Fortunately, a new generation of marker-free plasmids are now available which might promisingly replace the currently used plasmids such as pcDNA [10,11,12]. The nanoplasmid is designed based on an RNA-out system. These small sized plasmids harbor a very short (~500 bp) bacterial backbone consisting of a substantial sequence element of pUC origin of replication (R6K) and sucrose based RNA-out selection system. These small plasmids with a super-helical structure not only can satisfy the regulatory authorities but also can resolve the aforementioned technical problems intrinsic to large plasmids with big prokaryotic parts such as pcDNA and they are easily purified on a large scale.

Here we have compared the transfection efficiency and fluorescent protein expression in COS-7 cells transfected with pcDNA-EGFP (which works with antibiotic selection) and nanoplasmid-EGFP (which works with the RNA-Out system). The results indicated efficient transfection and expression with nanoplasmid-EGFP which makes it a promising alternative for human DNA vaccines. These results are especially attractive in respect to infections where a protective response is dependent on long-term Th1 immunity such as leishmaniasis.

## 2. Methods

### 2.1. Recombinant Plasmid Preparation

The pEGFP- N3 plasmid was digested with *Bam*HI-*Not*I enzymes to digest the EGFP gene (720 pb) and to clone it in pcDNA-3.1^+^ and nanoplasmid (NTC9385R-MSC which was a gift from Prof. Gerald Spaeth, Pasteur Institute of Paris) to generate pcDNA-EGFP (6148 bp) and NTC-EGFP (2473 bp) recombinants, respectively (Figure S1). Recombinant pcDNA-EGFP clones were selected on ampicillin containing LB-agar and recombinant NTC-EGFP clones were selected on 6% sucrose (Sigma, Taufkirchen, Germany) containing LB-agar (according to the manufacturer’s instructions). Plasmids from confirmed clones were purified with Endofree plasmid mega kit (Qiagen, Hilden, Germany).

### 2.2. Cell Line Culture

Adherent COS-7 cell line (fibroblast-like cell lines derived from monkey kidney tissue, ATTC: CRL-1651) was obtained from Iran National Institute of Genetic Engineering and Biotechnology and cultured in RPMI-1640 (Biowest, Nuaille, France) supplemented with 10% of heat inactivated FBS (hiFBS), 1% gentamicin, 1% HEPES (Sigma) and 1% L. glutamine (Sigma). Cells were seeded in 6 well plates (SPL, Pyeongtaek, South Korea) and cultured up to 70% confluency and then were sub-cultured within 2 days post-plating for adequate cell propagation.

### 2.3. Electroporation for Plasmid Transfer into Host Cells

On the day of electroporation, adherent COS-7 cells were harvested using 0.25% pre-warmed trypsin solution. Harvested cells were washed after neutralization with RPMI-1640 supplemented with 10% hiFBS. Cell pellets were re-suspended in PBS with pH adjusted to 7.2 and counted. A total of 10^6^ cells in 100 µL of PBS were distributed in 0.5 mL tubes, mixed with pcDNA-EGFP or nanoplasmid-EGFP and loaded in 2 mm electroporation cuvettes. Cells were electroporated with GenePulser Xcell^TM^ (Bio-Rad, California, USA) wave-square program (voltage of 180 mv, 2 waves with 0.1 s interval and pulse length of 5 ms). Immediately after pulsing, electroporated cells were plated in 6 well culture plates in antibiotic-free RPMI-1640. EGFP expression was monitored with fluorescent microscope on the following days (24 and 48 h after transfection). Plasmids were compared in equal (5 µg, 1 µg and 500 ng) and equimolar amounts (0.12 pmol, 0.24 pmol and 1.2 pmol). To reach equimolar concentrations, picomole of pcDNA-EGFP was calculated using the formula:

(1) $$\mathrm{pmol}=\mu g\;\left(dsDNA\right)\times \frac{10^{6}}{1\mu l}\times \frac{1\;\mathrm{pmol}}{660\mathrm{pg}}\times \frac{1}{\mathrm{bp}}$$

Then, NTC-GFP was used equimolar to pcDNA-GFP in each concentration.

### 2.4. Quantification of EGFP Expressing Cells with Flow Cytometry

The fluorescence level in transfected COS-7 cells was evaluated quantitatively by Flow cytometry. Cells were trypsinated and washed in PBS-1% Fetal Bovine Serum after harvesting from plates and re-suspended in the same buffer for data acquisition on CyFlow (Partec-Norderstedt, Germany) instrument. EGFP data was acquired on live cells and in FL1 channel. Data were analyzed with FlowJo version 7.5.3 (TreeStar, Ashland, USA) for percent of GFP positive cells and the MFI of GFP expression in FL1 channel. 

### 2.5. Quantitative Real Time PCR for Quantification of EGFP Copies in Transfected Cells

COS-7 cells transfected with both plasmids in equal concentration or equal moles were trypsinated and harvested 24 and 48 h after electroporation. RNA was extracted from each separate preparation with RNeasy^®^ plus mini kit (Qiagen) according to the manufacturer’s instructions. Purified total RNA was treated with DNase (2 u/500 ng RNA) to remove DNA remnants and final concentration was measured by nanodrop (Nanodrop ND1000). 100 ng of total RNA from each sample was then reverse transcribed to cDNA using a reverse transcription system according to the manufacturer’s instructions for RevertAid first strand cDNA synthesis kit (Thermo Fischer Scientific, Beijing, China). Two sets of primers were then used for quantitative RT-PCR reactions. PCR reactions were prepared including 400 nM of each GAPDH targeting forward (5′-TTCGAGAGTCAGCCGCATTT-3′) and reverse (5′-TTCCCGTTCTCAGCCTTCAC-3′) primers and 800 nM EGFP targeting forward (5′-ATCATGGCCGACAAGCAGAA-3′) and reverse (5′-TCTCGTTGGGGTCTTTGCTC-3′) primers in different tubes, 3µL cDNA, and 12.5µL Amplicone SYBR Green Master Mix in a total of 25 µL reaction mixture. The amplification cycles were programmed as follows: 1 cycle of 95 °C for 10 min; 40 cycles of 94 °C for 30 s, 60 °C for 30 s, and 72 °C for 30 s (Applied Biosystem 7500, Thermo Fischer Scientific, USA). The copy numbers of each target in unknown samples was extracted on pertaining standard curves for GAPDH and EGFP, respectively. The standard curves were plotted using cDNA samples of each target with 1:2 dilution factors. The Ct of each unknown sample was determined for each target individually and then the EGFP copy number was normalized against the GAPDH copy number in each individual sample. The copy numbers for each standard cDNA sample were internally assigned based on equivalent pmol of 100 ng starting mRNA.

### 2.6. Statistical Analysis

This experiment was conducted in two separate rounds. Data presented here are representative of the two experiments. Data were compared using Student’s *t*-test. All *p*-values are two-tailed. Statistical calculations were done in Graph-pad PRISM 9.0, Graphpad software Inc., California) and statistical significance is indicated with asterisks. * *p* < 0.05, ** *p* < 0.01, ***p < 0.001.

## 3. Results a

### 3.1. Nanoplasmid Efficiently Increases Protein Expression 

**Figure 1:**
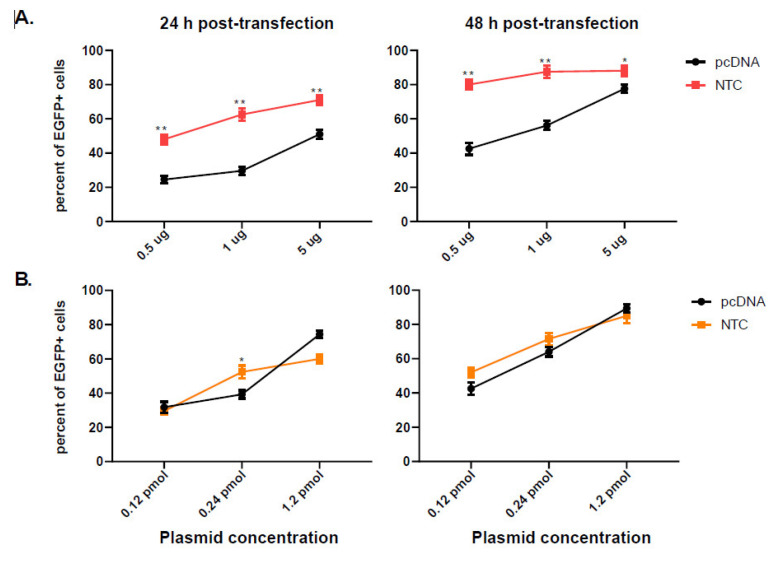
**EGFP positive COS-7 cells evaluated by flow cytometry.** COS-7 cells were transfected with equal concentration (**A**) or equimolar concentrations (**B**) of pcDNA-EGFP or NTC-EGFP. 24 h (left graphs) and 48 h (right graphs) later, cells were harvested and EGFP positive cells were analyzed by flow cytometry. 10,000 events were acquired by Partec instrument. The results are representative of two separate experiments; error bars indicate the within-assay repeats in each experiment. Data were compared using Student’s *t*-test. * *p* < 0.05, ** *p* < 0.01.

COS-7 cells were transfected with different concentrations of pcDNA-EGFP and NTC-EGFP (0.5 µg, 1 µg and 5 µg) to evaluate the EGFP expression by flow cytometry. As indicated in Figure 1A, 24 h post-transfection, the percentage of EGFP positive cells was significantly higher in NTC-EGFP transfected cells in all concentrations used. 48 h later, the expression level increased for both plasmids with NTC-EGFP running ahead of pcDNA-EGFP (*p*-value < 0.05). NTC-EGFP is almost one third of pcDNA-EGFP in size (2473 bp vs. 6148 bp) meaning that at equal concentrations, more NTC molecules are transfected than pcDNA molecules. 

In a separate experiment, NTC-EGFP and pcDNA-EGFP transfected cells at equimolar concentrations were also compared. In this case equal amounts of plasmids were transfected into COS-7 cells, and EGFP positive cells were evaluated 24 and 48 h later. As indicated in Figure 1B, 24 h post-transfection, a mild expression increase was observed in 1 µg NTC-EGFP transfected cells which is lost at higher concentrations. Further evaluation at 48 h, indicated no more significant increase especially at higher concentrations (5 µg). This indicates that NTC, at equimolar concentrations with pcDNA, almost transfects the cells with similar efficiency and with the same expression level. 

**Figure 2:**
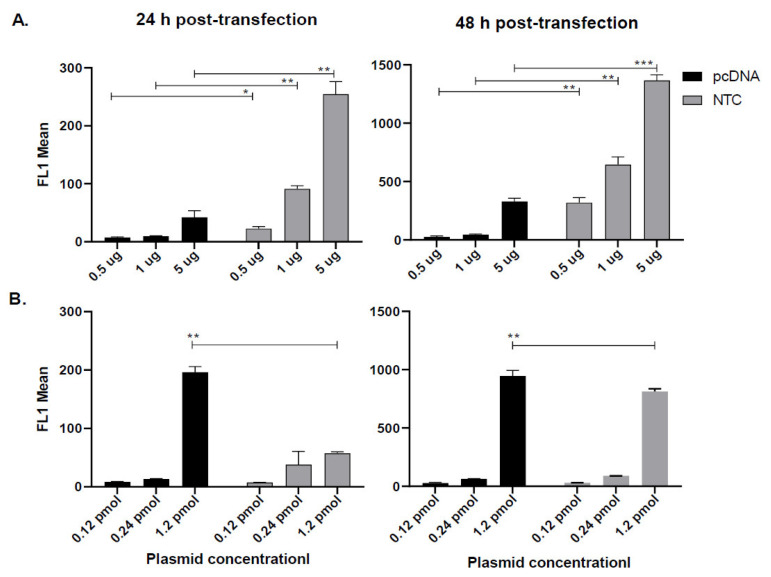
**MFI evaluated by flow cytometry.** COS-7 cells were transfected with equal concentration (**A**) or equimolar concentrations (**B**) of pcDNA-EGFp or NTC-EGFP. 24 h (left graphs) and 48 h (right graphs) later, cells were harvested and MFI was analyzed by flow cytometry in FL1 channel. A total of 10,000 events were acquired by the Partec instrument. The results are representative of two separate experiments and error bars indicate the within-assay repeats in each experiment. Data were compared using Student’s t-test. * *p* < 0.05, ** *p* < 0.01, *** *p* < 0.001.

Besides determining the percentage of GFP positive cells, we also evaluated the Mean Fluorescent Intensity (MFI) represented by the geometric mean, which indicates the average fluorescence level in all the cells transfected. Interestingly, at equal concentrations (Figure 2A), MFI was significantly higher in NTC-EGFP vs. pcDNA-EGFP transfected cells measured 24 and 48 h post-transfection (all concentrations examined). However, at equimolar concentrations (Figure 2B), although both plasmids acted similarly at 0.12 pmol and 0.24 pmol, NTC-EGFP transfection was significantly less than pcDNA transfection when the latter was at higher moles (1.2 pmol). This indicated that equivalent amounts of NTC and pcDNA plasmids are not equal in the expression profile of cells where higher concentrations of pcDNA skew the expression level towards higher MFIs. 

**Figure 3:**
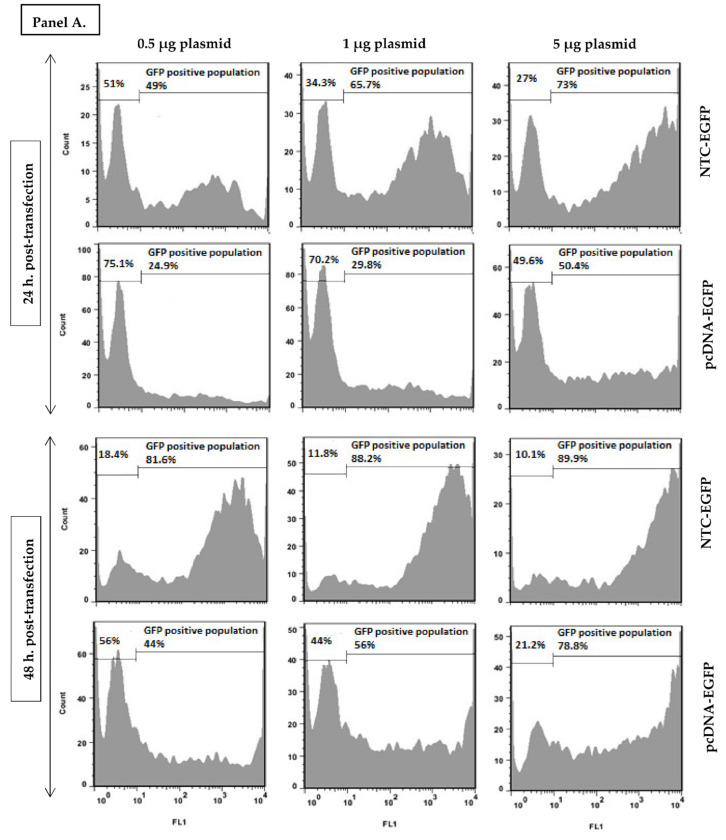

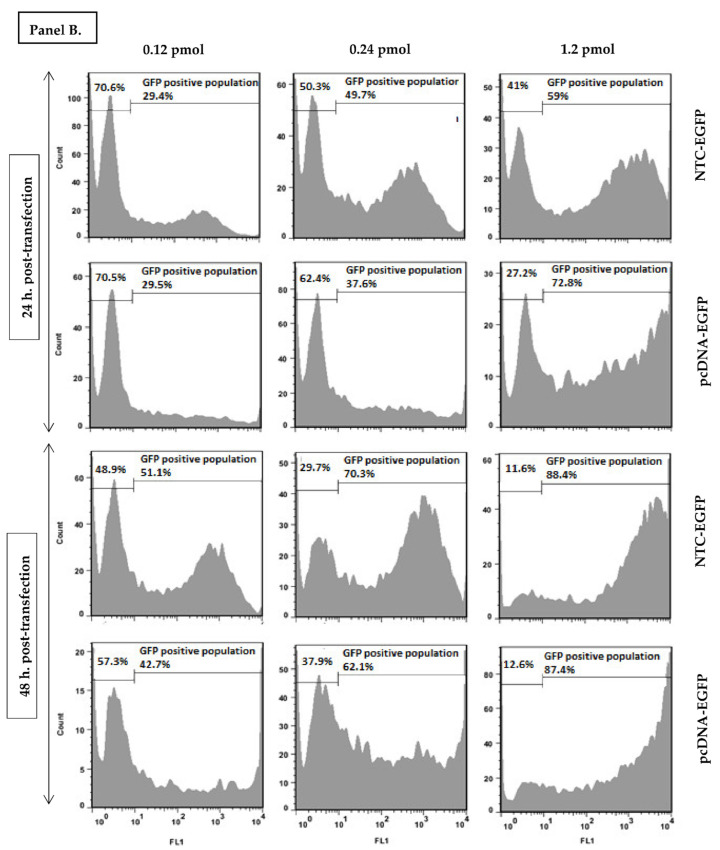
**Representative plots of NTC-EGFP and pcDNA-EGFP transfection.** EGFP positive population of COS-7 cells transfected with equal concentrations (Panel (**A**)) and equimolar concentration (Panel (**B**)) of NTC-EGFP and pcDNA-EGFP was evaluated by flow cytometry 24 h and 48 h post transfection.

As indicated in Figure 3, at equal concentrations (panel A), EGFP appears as early as 24 h post NTC transfection with less negative cells while many cells still remain negative at the time after pcDNA transfection. The same pattern can be observed even 48 h later. Interestingly, at equimolar concentrations, the plasmids are comparable at lower (but not higher) plasmid concentrations (Panel B). One remarkable note is the peak forming pattern of EGFP positive cells after NTC but not pcDNA transfection. Wherever cells are transfected with pcDNA, EGFP positive cells are distributed in a wide range of expression level from very low to very high quantities except in high concentrations (5 µg). Quite contrary, NTC transfected cells are distributed in a smaller range of fluorescence (in all concentrations used) resulting in a peak although not that sharp. This indicates that NTC transfected cells resemble each other more in expression level than pcDNA transfected cells. This phenomenon can in fact influence the outcome of DNA vaccination due to more similar expression levels among APCs receiving the NTC rather than pcDNA plasmid.

### 3.2. Conventional CMV Based pcDNA Generates More EGFP mRNA Copies

**Figure 4:**
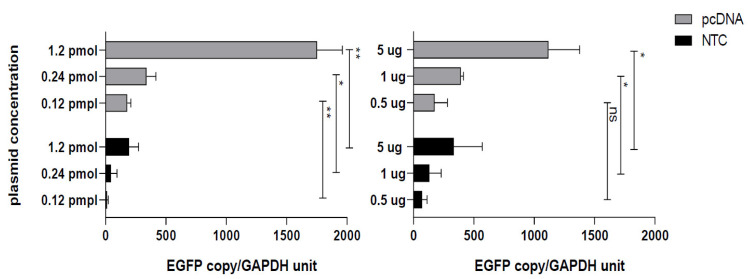
**Quantitation of EGFP mRNA level by q-PCR**. COS-7 cells transfected with NTC-EGFP or pcDNA-EGFP at equimolar (left panel) or equal concentration (right panel) were harvested 48 h post transfection for mRNA extraction. DNase treated mRNAs were subject to cDNA synthesis (100 ng as input mRNA). The relative quantities were extrapolated using standard curves obtained by EGFP and GAPDH serially diluted cDNAs. The results are representative of two separate experiments and error bars indicate the within-assay repeats in each experiment. Data were compared using Student’s t-test. * *p* < 0.05, ** *p* < 0.01. ns; non-significant.

In this study we compared the mRNA level by semi-quantitative q-PCR using two sets of primers targeting EGFP and GAPDH genes. As indicated in Figure 4, the EGFP mRNA level in pcDNA transfected cells significantly exceeds that in NTC transfected cells. At first glance, this is not in line with previous observations where NTC transfected cells and pertinent MFIs give higher results (at equal concentrations) or equal results (at equimolar concentrations) with pcDNA transfection. However if we refer back to the structure of NTC promoter, we will find a HTLV-I related region which promotes translation level but not the total mRNA level. This means that the lower mRNA level in NTC transfected cells is possibly compensated by higher translation at protein level when compared to a conventional CMV-based plasmid such as pcDNA. 

## 4. Discussion

Our data indicated that nanoplasmid (NTC 99385R) efficiently transfects COS-7 cells through electroporation. We compared this new generation of plasmids (harboring minimal bacterial backbone and additional enhancer elements inserted in the promoter sequence) with a commonly administered conventional CMV-based ampicillin dependent plasmid (pcDNA3.1^+^). Interestingly, equimolar concentrations (equal amount of molecules) of the two plasmids are comparable in respect to protein expression level (MFI) and transfection efficiency (positive populations) at lower plasmid concentrations but pcDNA goes beyond at higher molar concentrations. However, if the plasmids are compared at equal concentrations (different molecular amounts), since NTC is almost one third in size, it goes far ahead in all concentrations examined. We showed for the first time that although pcDNA derived target mRNA copies significantly exceed the NTC derived mRNA copies, the improved NTC promoter enhances the translation instead and increases protein production to comparable levels or even higher. 

First attempts to eliminate the unintended bacterial elements including selection markers and origin of replications resulted in capped DNA fragments called MIDGE. The bacterial backbone is excised by enzymatic digestion and the remaining portion with a eukaryotic promoter and target gene is re-ligated by adding 2 hairpin oligonucleotides making a dumbbell shaped vector. Unligated fragments are digested and MIDGE vectors are purified by anion-exchange chromatography. Schakowski et al. compared the in-vitro EGFP expression in chemically transfected cells with equal concentrations of EGFP-MIDGE vs. EGFP-uncut plasmid and found higher protein expression in EGFP-MIDGE transfected cells. If equimolar concentrations of EGFP-MIDGE vs. EGFP-uncut plasmid were compared no differences in transfection efficiency were observed in the analyzed cell lines but the amount of expressed target gene in cell supernatants was significantly higher in MIDGE transfected cells. This was the proof of concept that bacterial backbone can dramatically impact the eukaryotic expression of target genes [13]. In 2003, López-Fuertes et al. evaluated the protection conferred against *Leishmania (L.) major* induced infection using MIDGE encoding LACK protein with a NLS sequence. The protein expression in whole cell lysates of transiently transfected COS-7 cells with equimolar concentrations of MIDGE-Lack-NLS and pMOK-Lack (the parental plasmid) was evaluated by Western blotting. One day after transfection, the expression level of Lack protein was lower in MIDGE transfected cells but further immunization with equimolar plasmids similarly protected mice against an *L. major* infectious challenge. Most importantly, two doses of MIDGE-Lack-NLS conferred full protection similar to a pMOK-Lack prime/rVV-Lack boost, meaning that plasmids without a bacterial backbone could be efficiently substituted for viral vaccine delivery [14]. In 2014, another group evaluated the protective efficiency of a MIDGE vector encoding multiple immunogenic CD4 and CD8 inducing T cell epitopes from different *Leishmania* antigens (LEISHDNAVAX). Likewise, the nuclear translocation of the plasmid was enhanced using a NLS sequence. Three doses of this vector successfully protected against an *L. donovani* challenge with a Th1 skewed response [15,16]. As described, MIDGE constructs, although very effective in vivo, need a long complicated procedure for preparation. Moreover, they lose super-helical structure which is preferable for nucleic acid based vectors. For these reasons, minicircles were replaced [17]. The original plasmid contains two special recombination sequences flanking the target gene. Intramolecular recombination of a parental plasmid generates a minicircle containing the target gene and a miniplasmid containing the prokaryotic backbone. Therefore, fewer steps are needed to prepare a minicircle from a parental plasmid [18]. In many different studies, it has been shown that minicircles provide prolonged transgene expression in vivo and at equimolar quantities significantly reduce toxicity and increase immunogenicity relative to parenteral plasmid and could be advantageous for gene therapy or vaccine purposes [19,20]. Generally speaking, the production of MIDGE and minicircles is challenging since plasmid contaminants (especially remnants of plasmid backbone as miniplasmid) in minicircle preparations can be as high as 10% of the total yield—well beyond the 1.5% allowed by regulatory authorities. Therefore, production at industrial level still requires further optimizations.

Recently developed nanoplasmids can be more promising than MIDGE or minicircles with less preparation and purification complexities. Suschak et al. have published the results of nanoplasmid encoding antigens of VEEV and Ebola Virus used as vaccine. They have transfected COS-7 cells with 50, 100 and 250 ng of pWRG7077 conventional plasmid in parallel with equal concentrations of nanoplasmids encoding antigens from VEEV and Ebola Virus. Two days post-transfection, cells were harvested and analyzed by flow cytometry for surface expression of the antigens. At all different concentrations analyzed, nanoplasmid transfected cells expressed significantly higher levels of antigen. They further investigated the duration of expression up to 200 h post transfection with 50 ng of all different plasmids. The level of expression was significantly higher in nanoplasmid transfected cells even after 200 h post transfection. In vivo vaccination results further indicated that despite comparable levels of immune response in groups vaccinated with nanoplasmids or conventional pWRG7077 plasmid, the survival rate was significantly improved against VEEV or Ebola virus infectious challenge in nanoplasmid vaccinated groups. These results suggest that additional immunostimulatory elements on the nanoplasmid backbone such as CpG motifs and/or immunostimulatory small RNAs can further improve immunogenicity of nanoplasmids as non-viral vaccine vectors [21]. Borggren et al. have also compared the vaccination efficacy of pSSI conventional plasmid with nanoplasmids encoding a cocktail of 6 influenza vaccine antigens at equimolar concentrations and have found equal immunogenicity while measuring anti-HA antibody levels [22].

Interestingly, these new plasmid generations differ from the common ancestral CMV-based plasmids in promoter structure. This chimeric promoter incorporates additional elements besides CMV promoter/enhancer including the HTLV-I R-U5 sequence which contains the 5′-splice acceptor site and exon 2 splicing enhancer comprising the SR protein binding site (three copies of GAAGAAGAC) to improve translation and RNA export efficiency, respectively [23]. Barouch et al. explored the effects of adding the regulatory R region from the 5 long terminal repeat (LTR) of human HTLV-1, which acts as a transcriptional and posttranscriptional enhancer to CMV expression cassette. These new versions of DNA vaccines induced higher HIV-1 specific cellular immune responses compared to the original DNA plasmid (in equal concentrations) both in mice and monkeys. The R element actually potentiates expression from a strong CMV promoter/enhancer. Although the mechanism of action remains to be determined, it is likely that the R region activates key cellular transcription factors to enhance transcription and also functions as an internal ribosome entry site to enhance post-transcriptional translation [24]. To further evaluate the promoter effect, we analyzed the mRNA copies of EGFP produced in pcDNA-EGFP and NTC-EGFP transfected cells and surprisingly found less copies in NTC transfected cells at different plasmid concentrations used. We found no existing reports with similar results except for a study comparing the minicircles with the parental plasmid [19]. In this case, the mRNA level was higher in minicircles transfected cells using ΔΔCt and fold change analysis. Keeping in mind that the minicircle has the same promoter elements as the parental plasmid, we found that HTLV-1 enhanced promoter increases the target gene expression at the protein level and does not enhance total RNA level as described by nature biotechnology NTC9 series instruction manual (https://docplayer.net/90109323-Ntc9-series-nanoplasmid-tm-expression-vectorsinstruction-manual.html, Version 2 April 2018).

## 5. Concluding Remarks

DNA vaccines undoubtedly induce a robust and long lasting Th1 response and therefore are among the most effective subunit vaccine strategies against intracellular pathogens. However, regulatory authorities have hindered their general use in human societies. New generations of plasmids lacking the entirety or part of the bacterial backbone are now under investigation to replace the original plasmids. These new generations will resolve problems associated with plasmid uptake and migration to the nucleus and will improve eukaryotic prolonged target expression due to additional enhancer sequences of the promoter. They will fortunately satisfy regulatory authorities with a safe non-integrating and non-antibiotic dependent backbone which is easily produced and purified. These small-sized plasmids in equal or equimolar concentration with conventional plasmids have generated comparable or even higher immunogenicity in vivo. Some discrepancies between studies investigating equimolar concentrations in vitro can be explained by different cell types transfected using different strategies and by different technical methods used for protein expression detection. Our results promisingly indicated that the NTC9 series can successfully replace commonly used pcDNA vectors. The equimolar concentrations (with comparable in vitro results) or equal concentrations (with higher efficiency of the nanoplasmid in protein production) of the two plasmids can enter further vaccine studies to evaluate the protective efficiency of nanoplasmids compared to conventional plasmids. We still need to address some further questions such as whether plasmids without a bacterial backbone (e.g., MIDGE and minicircles) or with minimal sequences (e.g., the newly generated marker-free plasmids) need additional adjuvants for efficient immune stimulation if replacing conventional plasmids with potent innate immune stimulatory backbones.

## Data Availability

The data presented in this study are available in the article and Supplementary Materials.

## Supplementary Materials

The supporting information can be downloaded at: https://www.mdpi.com/article/10.3390/vaccines10101710/s1, Figure S1: Cloning map of NTC-EGFP and pcDNA-EGFP.
