# Supplementary figures and images for: Antibiotic-Free Nanoplasmids as Promising Alternatives for Conventional DNA Vectors
Source: Vaccines (Basel). 2022 Oct 13;10(10):1710. doi: 10.3390/vaccines10101710 (PMC9611672; doi:10.3390/vaccines10101710)

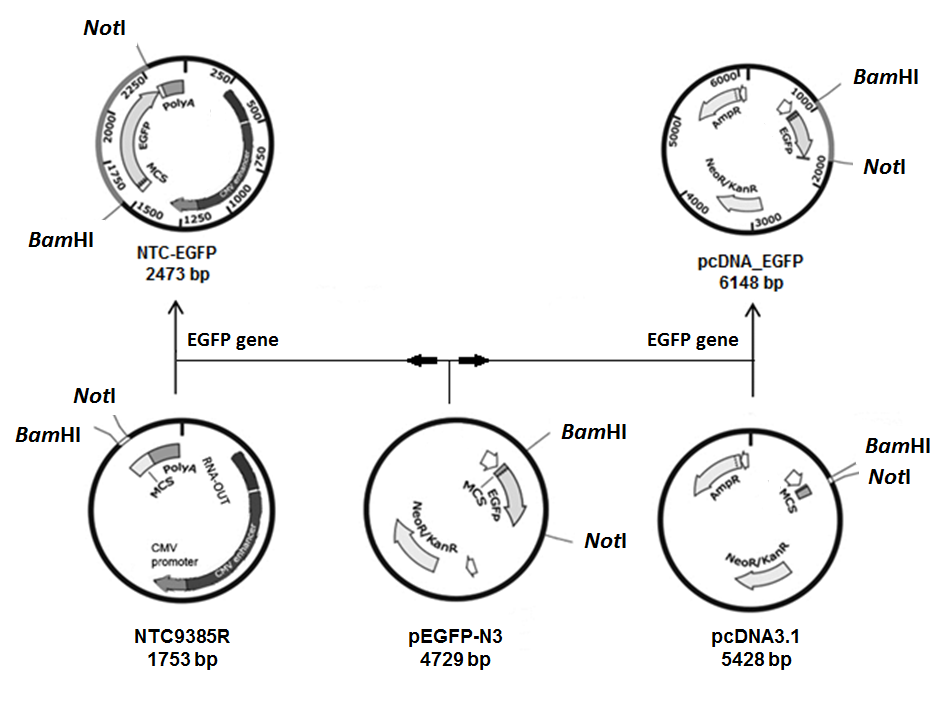

Supplement: Supplementary file 1 [file vaccines-10-01710-s001.zip › vaccines-1931201-supplementary.png]
